# Expanding the Genotype and Phenotype Diversity in a Chinese Cohort With TRPV4‐Related Dysplasia

**DOI:** 10.1111/cge.70103

**Published:** 2025-11-20

**Authors:** Lina Dong, Kwan Chun Ho, Zhijia Tan, Yanni He, Yapeng Zhou, Shijie Yin, Lin Feng, Janus Siu Him Wong, Michael Kai Tsun To

**Affiliations:** ^1^ Department of Orthopaedics and Traumatology The University of Hong Kong‐Shenzhen Hospital Shenzhen China; ^2^ Clinical Research Centre for Rare Diseases The University of Hong Kong‐Shenzhen Hospital Shenzhen China; ^3^ Translational Medicine Research Centre The University of Hong Kong‐Shenzhen Hospital Shenzhen China; ^4^ Department of Orthopaedics and Traumatology, Li Ka Shing Faculty of Medicine The University of Hong Kong Hong Kong Hong Kong

**Keywords:** genotype, phenotype, skeletal dysplasia, TRPV4‐Related dysplasia

## Abstract

Dominant mutations in the calcium permeable ion channel *TRPV4* (transient receptor potential vanilloid 4) typically result in skeletal dysplasia or peripheral neuromuscular disease. However, the full spectrum of *TRPV4*‐related phenotypes remains incompletely defined. This study systematically reviewed the clinical and genetic features of 10 Chinese patients harboring various *TRPV4* variants. In the cohort, six patients were diagnosed with spondylometaphyseal dysplasia Kozlowski type (SMDK) and four patients with metatropic dysplasia (MD). The most common features involved spinal deformity (platyspondyly, kyphosis or scoliosis), and lower‐limb malalignments (genu varum, genu valgum, or leg‐length discrepancy). Two patients with MD had neurological deficits. The R594H and P799R substitutions were the most recurrent variants in our study. A novel variant (c.1628T>G, p.L543R) in the S2‐S3 loop was identified. The study seeks to improve diagnostic precision by combining genetic and radiographic assessment, and highlights the importance of early spinal surveillance and multidisciplinary care to prevent neurological complications underlying *TRPV4*‐mediated disorders.

## Graphical abstract

Exploring the genotype and phenotype diversity in a Chinese cohort with TRPV4‐related dysplasia.

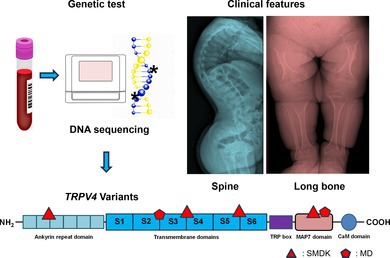

## 1 Introduction

TRPV4, a nonselective, calcium‐permeable cation channel, is widely expressed in neurons, kidney, lung, and bone [1]. Structurally, each TRPV4 comprises six transmembrane domains (S1–S6). Both the N‐ and C‐terminals are intracellular and contain functional domains, such as the ankyrin repeat domain (ARD) and TRP box [2]. Four TRPV4 subunits form a functional homo‐tetramer channel. Over 70 heterozygous missense mutations in *TRPV4* have been identified [3] associated with skeletal dysplasia and peripheral neuropathies. *TRPV4*‐related skeletal disorders span a phenotypic continuum, including lethal and nonlethal MD, parastremmatic dysplasia, spondyloepiphyseal dysplasia Maroteaux type (SEMD‐M), SMDK, autosomal dominant brachyolmia, and familial digital arthropathy brachydactyly (FDAB) [4]. *TRPV4*‐mediated neurodegenerative diseases include subgroups of hereditary motor and sensory neuropathy, congenital distal spinal muscular atrophy (CDSMA), scapuloperoneal spinal muscular atrophy (SPSMA), Charcot–Marie–Tooth disease type 2C (CMT2C) and more [4, 5, 6].

The exact mechanisms by which *TRPV4* mutations disrupt skeletal development remain incompletely understood. Most mutations result in a gain‐of‐function effect, characterized by increased basal channel activity, calcium influx, and reduction of sensitivity to small molecule antagonism [7, 8]. These alterations can impair endochondral ossification and matrix remodeling [9]. Interestingly, patients with *TRPV4* mutations show significant heterogeneity in skeletal and neuropathic syndromes. Some patients exhibit combined phenotypes [10], though no consistent genotype–phenotype correlation has been confirmed [7]. This variability and incomplete penetrance complicate diagnosis, prognostication and genetic counseling. Given these challenges, our study aims to systematically review the clinical presentations and radiographic features of patients with *TRPV4* mutations. By characterizing mutation‐specific phenotypes, this study seeks to improve diagnostic precision and contribute to the molecular mechanisms underlying *TRPV4*‐mediated disorders as well as targeted therapies.

## 2 Methods

### 2.1 Study Design and Data Collection

This study was approved by the Institutional Review Board of the University of Hong Kong–Shenzhen Hospital ([2020]190). Clinical, radiological and genetic data for patients diagnosed with *TRPV4*‐related skeletal dysplasia between 2020 and 2025 were retrospectively reviewed. Ten individuals (7 males and 3 females) were included.

### 2.2 Clinical and Radiological Assessment

Clinical information, including patient history, symptoms, and physical examination results, was systematically obtained. Radiographic features included: spinal deformities including scoliosis, platyspondyly, and overfaced pedicles (lateral vertebral edges extending beyond lateral pedicle borders [11]); pelvic configuration including halberd‐shaped pelvis (hypoplastic ilia with narrow sacrosciatic notches and crescent‐shaped iliac wings resembling a Sweden battle ax [12]), acetabular roof flattening and iliac‐wing morphology; long‐bone changes such as metaphyseal and epiphyseal abnormalities. MD was defined by dumbbell‐shaped long bones, prominent spinal and pelvic deformities. SMDK was defined by milder deformities without typical dumbbell‐shaped metaphyseal flaring. Final diagnoses were determined by pediatric orthopedic specialists after comprehensive review.

### 2.3 Genetic Test

With informed consent from adult patients or guardians (for patients age below 18), all patients underwent whole exome sequencing (WES) to detect the mutations in the *TRPV4* gene.

## 3 Results

### 3.1 Patient Characteristics and Initial Presentations

In this cohort, 10 patients were included, comprising 6 with SMDK and 4 with MD (Table 1). Detailed clinical features are summarized in the Supporting Information and Table S1. The mean onset age was 3 years old. The commonly reported initial clinical feature was spinal deformity, with platyspondyly, kyphosis or scoliosis. Additionally, short stature and gross motor developmental delay were frequently observed early manifestations, each occurring in approximately one‐third of the cohort. Furthermore, lower‐limb malalignments, such as genu varum, genu valgum, and leg‐length discrepancy, were also prevalent. Two patients with MD presented with neurological deficits secondary to spinal involvement.

**TABLE 1:** Summary of the clinical features of 10 patients with TRPV4 related disorders.

Case/gender	DOB	Diagnosis	Genotype	ACMG classification	Spine			Long bones			Pelvis		Neuron
					Scoliosis	Kyphosis/lordosis	Platyspondyly	Metaphyseal changes	Malalignment	Joint contracture	Abnormal pelvic	Acetabulum changes	Neuropathy
Case 1/M	Nov, 2011	SMDK	c.2396C>T, p.P799L	Pathogenic [22]	−	+	+	+	LLD, genu valgum, coxa valga	+	+	+	−
Case 2/F	Oct, 2017	SMDK	c.2396C>T, p.P799L	Pathogenic [22]	+	+	+	+	Genu valgum, coxa valga	+	+	+	−
Case 3/F	Dec, 2016	SMDK	c.1847G>A, p.R616Q	Pathogenic [25]	+	+	+	+	Mild genu valgum	+	−	−	−
Case 4/M	May, 2019	SMDK	c.1781G>A, p.R594H	Pathogenic [26]	+	−	+	+	LLD	+	−	−	−
Case 5/M	Apr, 2016	SMDK	c.1781G>A, p.R594H	Pathogenic [26]	+	−	+	+	Mild genu valgum, Coxa vara	+	+	+	−
Case 6/M	Aug, 2018	SMDK	c.694C>T, p.R232C	Pathogenic [24]	+	−	+	+	Genu valgum, coxa valga	+	+	−	−
Case 7/M	Oct, 2020	MD	c.1628T>G, p.L543R	VUS^a^	+	+	+	+	Coxa valga	+	+	+	−
Case 8/M	May, 2005	MD	c.2389G>A, p.E797K	Pathogenic [22]	+	+	+	+	Genu valgum, LLD	+	+	−	−
Case 9/F	Jul, 2015	MD	c.2396C>G, p.P799R	Pathogenic [26]	+	+	+	+	LLD	+	+	+	+
Case 10/M	May, 2018	MD	c.2396C>G, p.P799R	Pathogenic [26]	+	+	+	/	/	/	/	/	+

Abbreviations: LLD, leg length discrepancy; MD, metatropic dysplasia; SMDK, spondylometaphyseal dysplasia Kozlowski type; VUS, variant of uncertain significance.

^a^ According to the Standards and Guidelines for the Interpretation of Sequence Variants from the American College of Medical Genetics (ACMG), this variant was classified as a variant of uncertain significance (VUS) based on the rules for combining criteria to classify sequence variants (PM6 + PP2 + PP4).

### 3.2 Spinal Features

**FIGURE 1:**
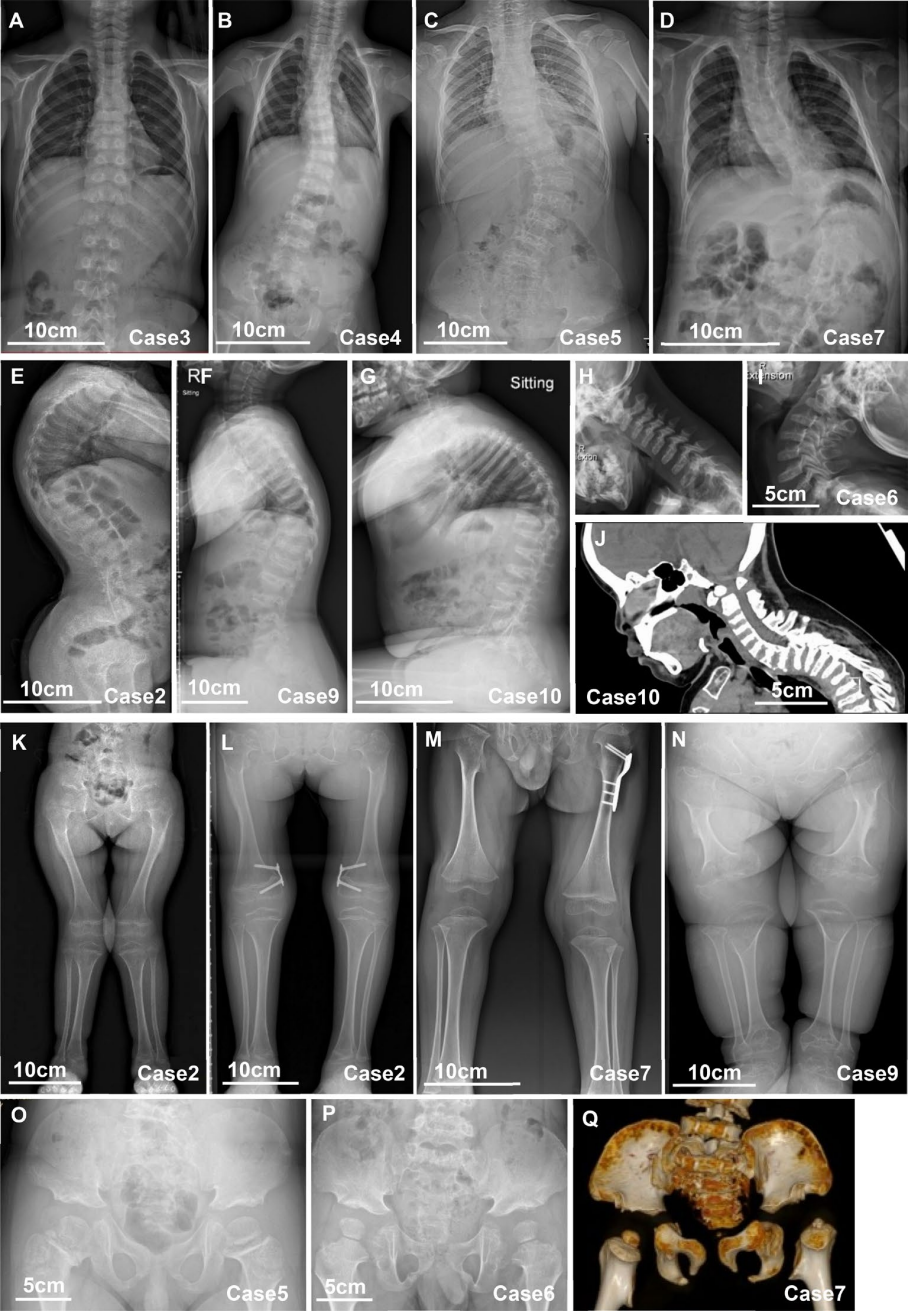
Skeletal deformities of the cohort. (A–D) Scoliosis with variability ranging from mild to severe. (E–G) Thoracic hyper‐kyphosis, lordosis and platyspondyly. (H–I) Atlantoaxial instability with posterior translation of C3–C5 on extension imaging. (J) Posterior translation of the T2‐T5 segments. (K–L) Genu valgum and postoperation follow‐up (2 years): Bilateral eight‐plate hemi‐epiphyseal block. (M) Leg‐length discrepancy. (N) Dumbbell‐shaped metaphyseal flaring. (O–Q) Mildly halberd‐shaped pelvis and flattened acetabular roofs.

Scoliosis was highly prevalent, identified in 9/10 patients (90%), with considerable variability in severity. Some developed progressive scoliosis with Cobb angles exceeding 45° as early as 1 to 2 years old, while others exhibited mild curves (10°–20°) without progression (Figure 1A–D). Hyper‐kyphosis or hyper‐lordosis was observed in 7/10 patients (70%), including all MD patients and half of SMDK patients (Figure 1E–G). Platyspondyly was among the most consistent radiographic findings. Overfaced pedicles were present in 6/10 patients (60%). Atlantoaxial instability and spinal stenosis were identified in 5/10 patients (50%) (Figure 1H–J). Notably, two MD patients presented with spinal cord compression with neurological deficits, whereas none of the SMDK patients exhibited neurological complications.

### 3.3 Long‐Bone Abnormalities

Variable metaphyseal enlargement was observed in 9/9 patients with available imaging (100%) (no proper lower limb X‐ray images for Case 10) (Figure 1K–N). Dumbbell‐shaped metaphyseal flaring was the characteristic feature of MD patients (Figure 1M,N), whereas SMDK patients typically displayed subtle or mild flaring (Figure 1K,L). Epiphyseal dysplasia was identified in 3/9 patients (33.3%). Leg‐length discrepancy (LLD) occurred in 4/9 patients (44.4%), ranging from 8 mm to 31 mm (Figure 1M). Lower‐limb malalignment was present in all documented cases, most frequently in the form of genu valgum. 3/9 patients (33.3%) underwent corrective procedures, such as guided growth (temporary hemiepiphysiodesis) with tension band plates. One case achieved a successful outcome (Figure 1K,L). Joint contractures and restricted ranges of motion were documented in 9/9 patients (100%), affecting both upper and lower extremity joints. Waddling gait was noted in 3/9 patients (33.3%). Given the absence of lower‐limb weakness or sensory deficits on clinical examination, this gait abnormality was likely attributable to mechanical factors, such as hip deformity, joint contractures or joint instability, rather than neuromuscular dysfunction.

### 3.4 Pelvic Morphology

Regarding pelvic morphology, a halberd‐shaped pelvis was observed in 7/9 patients with available imaging (no proper pelvis X‐ray images for Case 10) (77.8%, Figure 1O–Q). The deformity was typically more pronounced in MD patients, while often milder or absent in SMDK cases. Flattened acetabular roofs occurred in 5/9 patients (55.6%, Figure 1O–Q). Iliac‐wing abnormalities, including shortening and broadening of the ilium, were present in 7/9 patients (77.8%).

### 3.5 Genotypes Presentation

**FIGURE 2:**
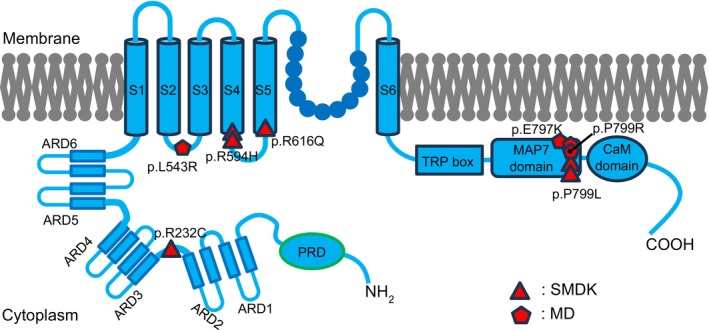
Disease causing mutations in TRPV4 with domain structure. A schematic view of TRPV4 channel indicating the variants identified in our cohort that cause SMDK and MD. ARD: ankyrin repeat domain; CaM: calmodulin; MAP7: microfilament‐associated Protein 7; PRD: proline‐rich domain; S1–S6: transmembrane segments.

In our study, three distinct genotypes were identified among the MD patients (Table 1, Figure 2). One patient carried the c.1628T>G variant (L543R substitution in the S2‐S3 loop), which represents a novel variant not previously reported. According to the ACMG guidelines, this variant was classified as VUS (variant of uncertain significance) (Table 1). The second patient harbored the c.2389G>A (E797K) mutation and the last two MD cases carried the c.2396C>G (P799R) transition located in the MAP7 domain [13]. Among the SMDK patients, four different genotypes were detected (Table 1, Figure 2). The c.694C>T (R232C) mutation was identified in the loop between the ARD2‐3 [14]. One patient carried the c.1847G>A (R616Q) variant in the S5 transmembrane domain [15]. Another two patients harbored the c.1781G>A (R594H) transition between S4 and S5 in the cytoplasmic region [16]. Lastly, two patients had the c.2396C>T (P799L) mutation in the MAP7 domain [16].

## 4 Discussion

### 4.1 Genotype–Phenotype Correlations

In this study, several genotypes were identified, including c.1781G>A (R594H) for SMDK and c.2396C>G (P799R) for MD, consistent with previously reported recurrent variants [13]. However, given the small, single‐center cohort (*n* = 10) and potential ascertainment bias, larger multicenter studies are needed to define the global distribution and frequency of these variants, and their association with clinical phenotypes. A number of patients harbored mutations typically associated with alternate skeletal dysplasia or neuropathy. Notably, two SMDK patients carried the c.2396C>T (P799L) mutation, a variant previously recognized as a common pathogenic mutation for MD [1, 7]. Both patients displayed mild‐to‐moderate skeletal features more consistent with SMDK rather than classic MD. Another SMDK patient had the c.1847G>A (R616Q) mutation linked to autosomal dominant brachyolmia type 3 [15]. One SMDK patient involved the c.694C>T (R232C) variant previously reported as associated with hereditary motor and sensory neuropathy and congenital distal spinal muscular atrophy [14, 17]. In the MD group, one patient carried a novel c.1628T>G (L543R) mutation, identified in prior studies as related to Wolfram syndrome, an autosomal recessive neurodegenerative disorder [18], but not previously linked to *TRPV4*‐related skeletal disease. The phenotype diversity of *TRPV4*‐related skeletal dysplasia and peripheral neuropathy likely reflects the levels of *TRPV4* activation caused by different genetic variants. That is why mutations within the same TRPV4 domain can lead to such different disease phenotypes [4]. It is reported that the mutation of c.1846C>G (R616G) in *TRPV4* caused a severe neuropathy phenotype and bilateral vocal cord paralysis, while the c.1847G>A (R616Q) substitution in the same amino acid only led to isolated skeletal dysplasia [8], consistent with our study. These findings consistently suggest a lack of clear genotype–phenotype correlation within the *TRPV4* mutation spectrum. They underscore the importance of combining genetic testing with detailed clinical evaluation, as genetic results alone may not reliably distinguish between MD and SMDK.

### 4.2 Spinal Instability and Neurological Complications

Spinal cord compression and neurological deficits are among the most serious complications of *TRPV4*‐related dysplasia, typically resulting from spinal canal stenosis and atlantoaxial instability [19]. In our cohort, atlantoaxial instability was identified in 5 patients (50%), and spinal stenosis, affecting either the cervical or thoracic spine, in 3 patients (30%), spanning both MD and SMDK groups. While most SMDK patients remained asymptomatic, MD patients were more likely to exhibit significant neurological manifestations, suggesting that the degree of baseline calcium elevation caused by *TRPV4* mutations correlates with the development of mixed phenotypes in skeleton and peripheral nerves [8]. These findings underscore the importance of routine spinal surveillance in patients with *TRPV4*‐related dysplasia. Careful neurological examination and MRI of the spine should be included in long‐term follow‐up, even for asymptomatic individuals. Flexion‐extension cervical films should be performed to allow early detection of atlantoaxial instability, as previously recommended [20]. Early identification of spinal instability or stenosis enables timely surgical intervention, preventing irreversible neurological damage. In our series, one patient who underwent cervical spinal fusion shortly after the onset of weakness showed partial neurological recovery, whereas another patient who received surgery after near‐complete paralysis had no postoperative neurological recovery. These contrasting outcomes highlight the importance of timely diagnosis and intervention. Moreover, it is essential to differentiate *TRPV4*‐related peripheral neuropathy from neurological abnormalities secondary to vertebral compression, as the two require distinct management approaches.

For conservative treatment, spinal deformities tended to progress rapidly. Early brace applications can help slow progression during the flexible stage. When bracing fails to control progression, body casting may be required to prevent further deterioration. For surgical management, the approach should depend on the degree of instability and neurological involvement. In patients with cervical instability or spinal stenosis without cord compression, fusion without decompression may be adequate. Patients with cord compression, significant neurological deficits, or progressive symptoms despite conservative treatments should undergo decompression with fusion. Further longitudinal studies are required to refine management protocols and establish evidence‐based clinical practice guidelines.

### 4.3 Functional Impairment and Role of Multidisciplinary Approach

Apart from skeletal deformities, functional impairments such as restricted joint motion, joint contractures, and motor developmental delays were frequently observed in our cohort. These challenges can significantly impact patients' functional independence and quality of life, particularly during childhood in school. As such, multidisciplinary management is essential for patients with *TRPV4*‐pathy. In addition to pediatric orthopedic specialists, the care team should include pediatricians to monitor developmental milestones and identify comorbidities; physiotherapists to guide range of motion and muscle strengthening exercises; occupational therapists to design orthotics, and support home environment adaptation; mental health professionals to assess the psychosocial impact on both patients and their families; and social workers to facilitate access to educational and financial resources. Early and individualized rehabilitation, provision of mobility aids, and coordinated family support may reduce long‐term disability and promote more holistic physical and psychosocial development of the patients.

## Disclosure

The authors have nothing to report.

## Supporting information


**Table S1:** Supplementary clinical features of 10 patients with TRPV4 variants and related disorders.

## Data Availability

The data that supports the findings of this study is available in the Supporting Information S1 of this article.
