# Supplementary material for: Expanding the Genotype and Phenotype Diversity in a Chinese Cohort With TRPV4‐Related Dysplasia
Source: Clin Genet. 2025 Nov 20;109(4):796–802. doi: 10.1111/cge.70103 (PMC12958006; doi:10.1111/cge.70103)
Supplement: Supplementary file 1 — Table S1: Supplementary clinical features of 10 patients with TRPV4 variants and related disorders. [file CGE-109-796-s001.pdf]

Table S1. Supplementary clinical features of 10 patients with TRPV4 variants and related disorders

Case / Gender	DOB	Diagnosis	Age at onset	Interventions		
				Non-surgical intervention	Surgical intervention	Outcome
Case 1 / M	Nov, 2011	SMDK	10 years old	-	-	/
Case 2 / F	Oct, 2017	SMDK	1.4 years old	Spinal brace	Bilateral hemiepiphysiodesis	Improved LL alignments, Scoliosis progression halted
Case 3 / F	Dec, 2016	SMDK	1.5 years old	-	-	/
Case 4 / M	May, 2019	SMDK	At birth	-	-	/
Case 5 / M	Apr, 2016	SMDK	1 year old	Spinal brace	-	Progressive scoliosis despite bracing
Case 6 / M	Aug, 2018	SMDK	3 years old	Spinal brace	Bilateral hemiepiphysiodesis	Progressive scoliosis despite bracing
Case 7 / M	Oct, 2020	MD	2 years old	Spinal brace, Body cast	Subtrochanteric femoral osteotomy	Improved kyphotic deformity, hip subluxation reduced
Case 8 / M	May, 2005	MD	8 years old	-	Femoral osteotomy, Unilateral hemiepiphysiodesis	Improved LL alignments
Case 9 / F	Jul, 2015	MD	1 year old	-	Posterior atlantoaxial fusion	Improved lower-limb strength
Case 10 / M	May, 2018	MD	1 year old	-	Osteotomy, spinal fusion with internal fixation	Persistent neurological deficits

SMDK: spondylometaphyseal dysplasia Kozlowski type; MD: metatropic dysplasia; LL: leg length; VUS: variant of uncertain significance.

Supplementary-detailed case presentation

Case 1 / Male (SMDK, c.2396C>T (p.P799L))

Individual 1 clinically presented with genu valgum. Spinal imaging demonstrated irregular vertebral bodies, platyspondyly, overfaced pedicles, and mild thoracic hyper-kyphosis. Long-bone radiographs showed mild metaphyseal flaring of the femora and tibiae and a 10 mm leg-length discrepancy (right shorter than left). Pelvic imaging revealed a mildly halberd-shaped configuration with flattened acetabular roofs and short, broad iliac wings. No neurological, respiratory, or hearing problems were reported.

Case 2 / Female (SMDK, c.2396C>T (p.P799L))

Prenatal ultrasound revealed shortened limbs. During early childhood, she presented with short stature and progressive thoracolumbar kyphosis, for which a spinal brace was prescribed. Gross-motor delay was evident which she could walk independently but required assistance to climb stairs by age five. Spinal imaging demonstrated platyspondyly and mild scoliosis (Cobb angle 11° between L1 and L5). Long-bone radiographs showed mild bowing of the femora, metaphyseal flaring of the femora and tibiae, and coxa valga. Pelvic imaging revealed a mildly halberd-shaped configuration with flattened acetabular roofs and short, broad iliac wings. She subsequently underwent bilateral hemiepiphysiodesis with eight-plates at the medial distal femur to correct the progressive deformity. Two years postoperatively, the left lower limb achieved normal alignment, while the right retained mild valgus. She remained neurologically intact, with no respiratory or auditory symptoms.

Case 3 / Female (SMDK, c.1847G>A (p.R616Q))

Individual 3 clinically presented a high-arched back with lumbar hyper-lordosis and anterior pelvic tilt at the age of 4, which progressively became more pronounced. Physical examination revealed joint contractures with limited extension in the hips and elbows, and genu valgum became evident during later childhood. Spinal imaging

demonstrated mild scoliosis with lateral displacement from T6 to L3 and Cobb angle 10°. Long-bone radiographs showed mild metaphyseal flaring. The pelvis appeared grossly normal. She reported no neuropathy, respiratory difficulties, or hearing impairment.

Case 4 / Male (SMDK, c.1781G>A (p.R594H))

Individual 4 presented with genu varum and scoliosis at birth, followed by the development of a waddling gait at the age of 3. Spinal radiographs showed scoliosis with a Cobb angle of 26° between T7 and L2 and platyspondyly. Long-bone imaging revealed mild metaphyseal flaring and a LLD of 8 mm (left shorter than right). The pelvis appeared grossly normal. He reported no neurological deficits, respiratory difficulties, or hearing impairment.

Case 5 / Male (SMDK, c.1781G>A (p.R594H))

Individual 5 presented with easy fatigability during walking and a left-sided rib hump on forward bending at the age of 1, Genu varum noted at age of 3 gradually progressed to genu valgum, and spinal deformity became apparent by age of 4. A spinal brace was prescribed but proved ineffective. Spinal imaging demonstrated platyspondyly, bullet-shaped vertebral bodies, overfaced pedicles, and rotation scoliosis of lumbar spine. The thoracolumbar Cobb angle increased from 27° at age 2 to 45° at age 8, indicating significant curve progression. Long-bone radiographs showed mild metaphyseal flaring at the distal femur. Pelvic imaging showed a mildly halberd-shaped configuration, with mild acetabular and iliac-wing dysplasia. He reported no neurological deficits, respiratory difficulties, or other systemic abnormalities.

Case 6 / Male (SMDK, c.694C>T (p.R232C))

Individual 6 presented with short stature since birth, joint contractures with limited hip flexion and external rotation, and scoliosis. A spinal brace was prescribed at the age of

2, but the curvature progressed, and the spinal deformity became more pronounced by age of 5. Spinal radiographs showed irregular vertebral bodies with anterior wedge-shaped deformities, platyspondyly, atlantoaxial instability with posterior translation of C3-C5 during neck extension. Long-bone imaging revealed bilateral metaphyseal flaring of the femora and tibiae and prominent genu valgum. Pelvic radiographs showed mild halberd-shaped configuration. He underwent bilateral hemiepiphysiodesis with eight-plates for correction of genu valgum. He remained neurologically intact, with no respiratory or auditory abnormalities.

Case 7 / Male (non-lethal MD, c.1628T>G (p.L543R))

Individual 7 presented with kyphoscoliosis at 8 months of age. A spinal brace was prescribed initially, but the curvature continued to progress, resulting in a pronounced deformity. A body cast was subsequently applied which proved effective and led to significant improvement in the kyphotic deformity. Spinal imaging demonstrated S-shaped scoliosis with a 45° right-sided thoracic curve and a 48° left thoracolumbar curve, along with severe thoracolumbar hyper-kyphosis, platyspondyly, overfaced pedicles and atlantoaxial instability. Long-bone imaging revealed coxa valga and dumbbell-shaped metaphyseal flaring in the humerus, distal femur, and proximal tibia. Pelvic imaging showed a mild halberd-shaped configuration with horizontal acetabular roofs and short, broad iliac wings. Hip subluxation was noted and treated with open reduction, subtrochanteric femoral osteotomy, and adductor and iliopsoas tenotomy. He remained neurologically intact, with no respiratory or auditory abnormalities.

Case 8 / Male (non-lethal MD, c.2389G>A (p.E797K))

Individual 8 presented at the age of 8 with short stature, genu valgum, scoliosis, a waddling gait, and leg-length discrepancy. Spinal imaging revealed mild scoliosis with a Cobb angle of 16° in the thoracic segment, mild kyphosis, platyspondyly and overfaced pedicles. Long-bone imaging showed dumbbell-shaped metaphyseal flaring, epiphyseal dysplasia, femora dysplasia with flattened femoral heads and shortened, thickened femoral necks, genu valgum, 31mm LLD (right shorter than left), and osteophyte formation at knee joints. Pelvic radiographs demonstrated a mildly halberd-shaped configuration with broad and short iliac wings. He underwent a right distal femoral osteotomy and left-sided temporary hemiepiphysiodesis with eight-plate for the correction of lower-limb malalignment. He remained neurologically intact, with no respiratory or auditory symptoms.

Case 9 / Female (non-lethal MD, c.2396C>G (p.P799R))

Individual 9 presented with short stature at the age of 1, later developing hip flexion contractures, restricted hip and knee extension, and limited upper-limb range of motion. Spinal radiographs showed mild scoliosis with Cobb angle 18° from T4 to T12, thoracic hyper-kyphosis, lumbar lordosis, atlantoaxial instability platyspondyly, irregular vertebral bodies, and overfaced pedicles. Long-bone imaging showed dumbbell-shaped metaphyseal flaring of the humeri and femora, bilateral dislocated shoulder joints, and a 14mm LLD (right side shorter than left). Pelvic radiographs demonstrated a halberd-shaped configuration with broad, short iliac wings and narrow sacro-sciatic notches, and mild acetabular dysplasia. Her atlantoaxial instability progressed to spinal compression, resulting in bilateral lower-limb weakness, more pronounced on the right, along with gait difficulties. She underwent posterior atlantoaxial fusion, which led to improved muscle strength. She had no respiratory and hearing problems.

Case 10 / Male (non-lethal MD, c.2396C>G (p.P799R))

Individual 10 presented with short lower limbs, gross motor delay, bilateral hip dislocation and spinal kyphosis at the age of 1. Neurological examination revealed bilateral lower-limb weakness, sensory loss, and urinary and fecal incontinence, accompanied by decreased abdominal reflexes, hyperreflexia of the lower limbs, ankle clonus, and a positive Babinski sign. Spinal radiographs demonstrated S-shaped scoliosis with a 21° rightward curvature in the upper thoracic region and a 23° leftward deviation from T5 to L4, reduced cervical lordosis and thoracic hyper-kyphosis, severe platyspondyly, overfaced pedicles, atlantoaxial instability with C1-C2 anterior displacement on flexion, along with posterior translation of the T2-T5 segments resulting in spinal canal stenosis and spinal cord compression. He underwent posterior spinal scoliosis correction with osteotomy, spinal fusion with internal fixation, spinal canal decompression, and neural foramen decompression at the age of 6. Despite surgery, his neurological deficits persisted, with lower limb paralysis, sensory loss below the T2 level, areflexia of knee and ankle jerks. Arrhythmia subsequently developed as well. He had no respiratory or hearing difficulties.

Supplementary-Case 1

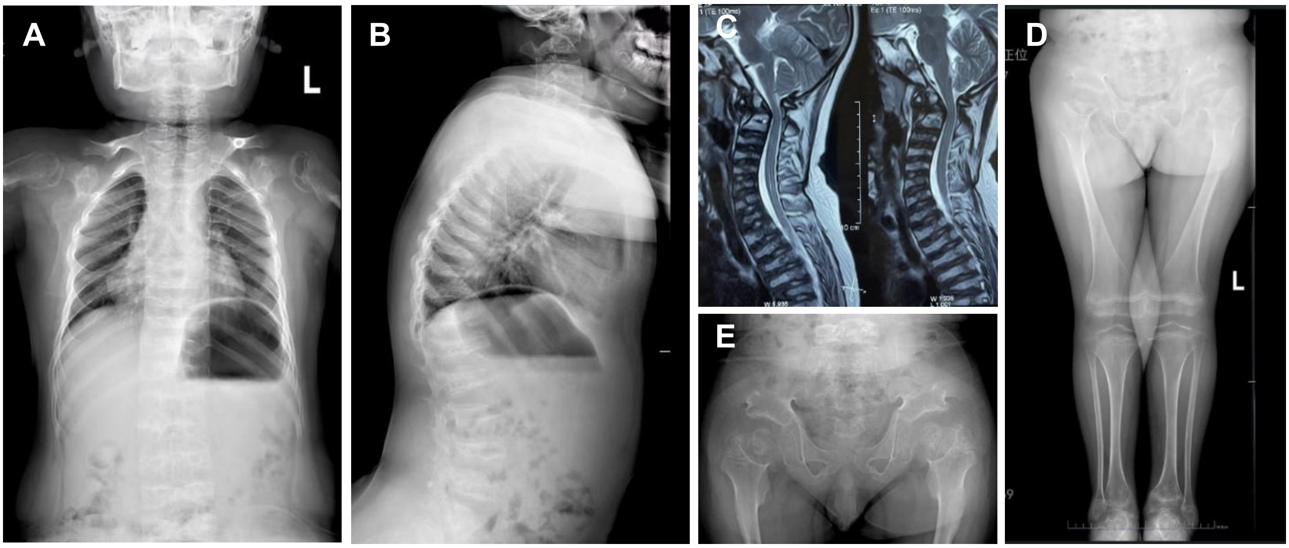

Clinical features of Case 1.

(A-B) X-ray showed thoracic hyper-kyphosis. **(C)** CT scan showed cervical spinal stenosis. **(D)** Metaphyseal flaring of the femora and tibiae, genu valgum, coxa valga. **(E)** Mildly halberd-shaped pelvis.

Supplementary-Case 2

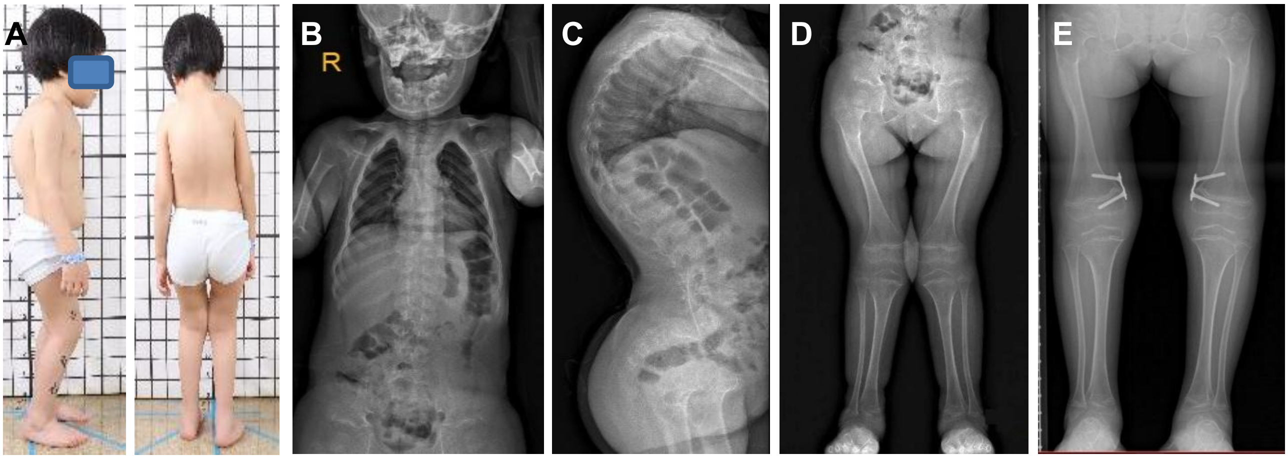

Clinical features of Case 2.

(A) Clinical photos of Case 2. **(B-C)** Spine: mild scoliosis, thoracic hyper-kyphosis, platyspondyly. **(D)** Long bones: genu valgum, metaphyseal flaring of femurs and tibiae. **(E)** Post-operation follow-up (2 years): bilateral eight-plate hemi-epiphyseal block.

Supplementary-Case 3

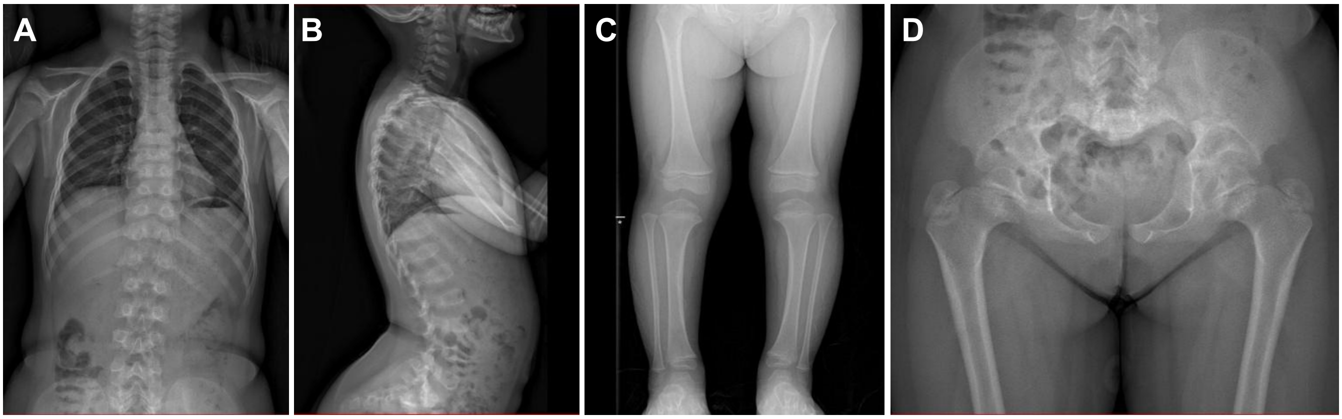

Clinical features of Case 3.
(A-B) Spine: mild scoliosis. **(C)** Long bones: mild metaphyseal flaring. **(D)** Pelvis: normal development.

Supplementary-Case 4

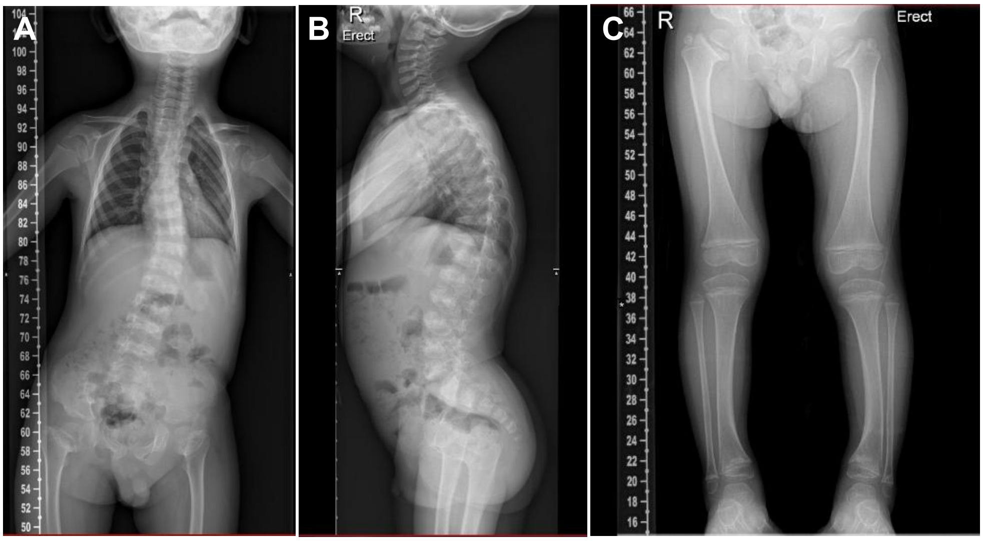

Clinical features of Case 4.
(A-B) Spine: platyspondyly, scoliosis. **(C)** Long bones: mild metaphyseal flaring.

Supplementary-Case 5

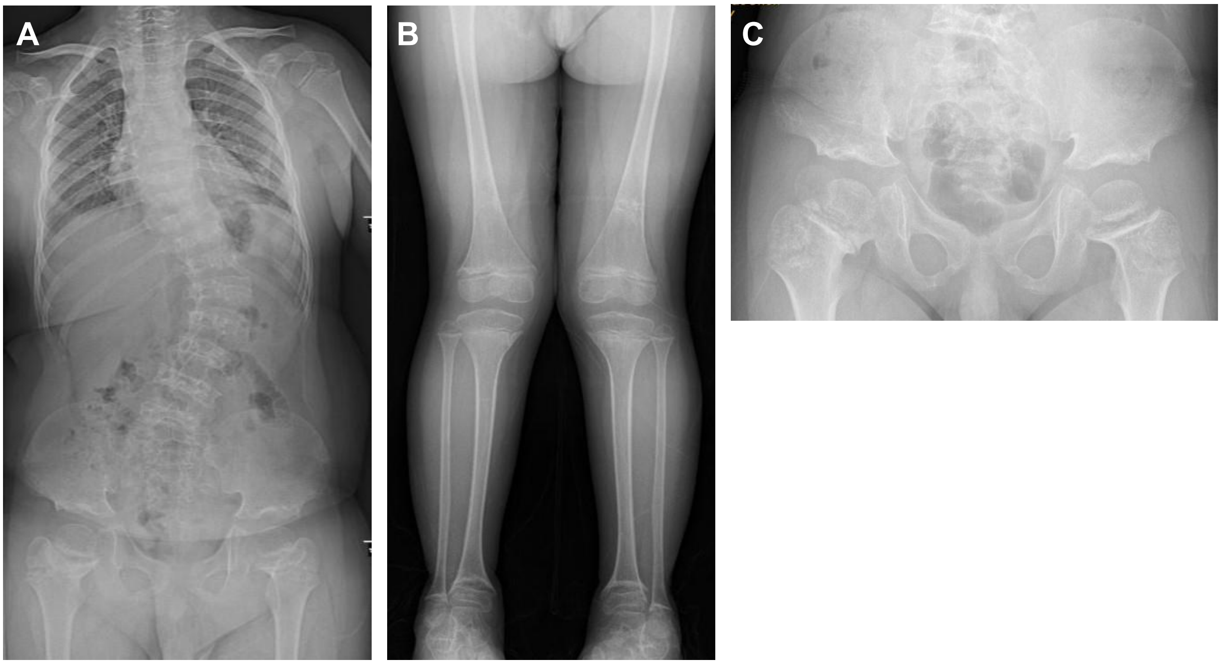

Clinical features of Case 5.

(A) Spine: platyspondyly, scoliosis, overfaced pedicles. **(B)** Long bones: mild metaphyseal flaring. **(C)** Pelvis: mildly halberd-shaped pelvis.

Supplementary-Case 6

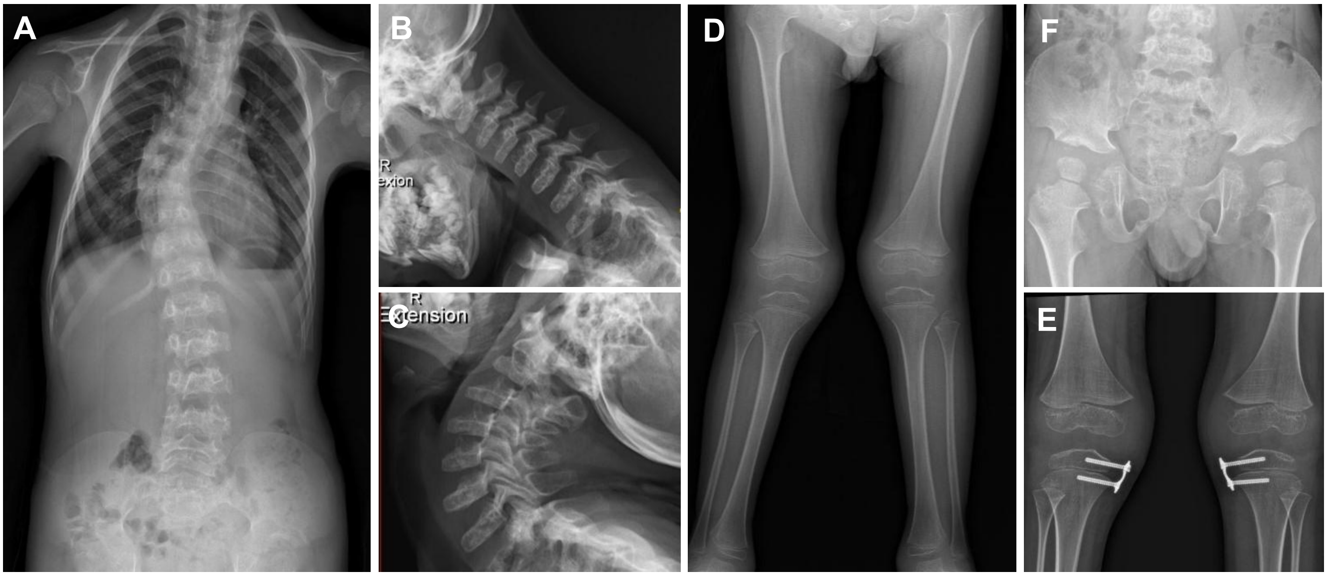

Clinical features of Case 6.

(A-C) Spine: S-shaped scoliosis, platyspondyly, rotational deformity, atlantoaxial instability with posterior translation of C3-C5 on extension imaging. **(D)** Long bones: metaphyseal flaring of the femora and tibiae, genu valgum. **(E)** Post-bilateral eight-plate hemi-epiphyseal block. **(F)** Pelvis: mildly halberd-shaped pelvis.

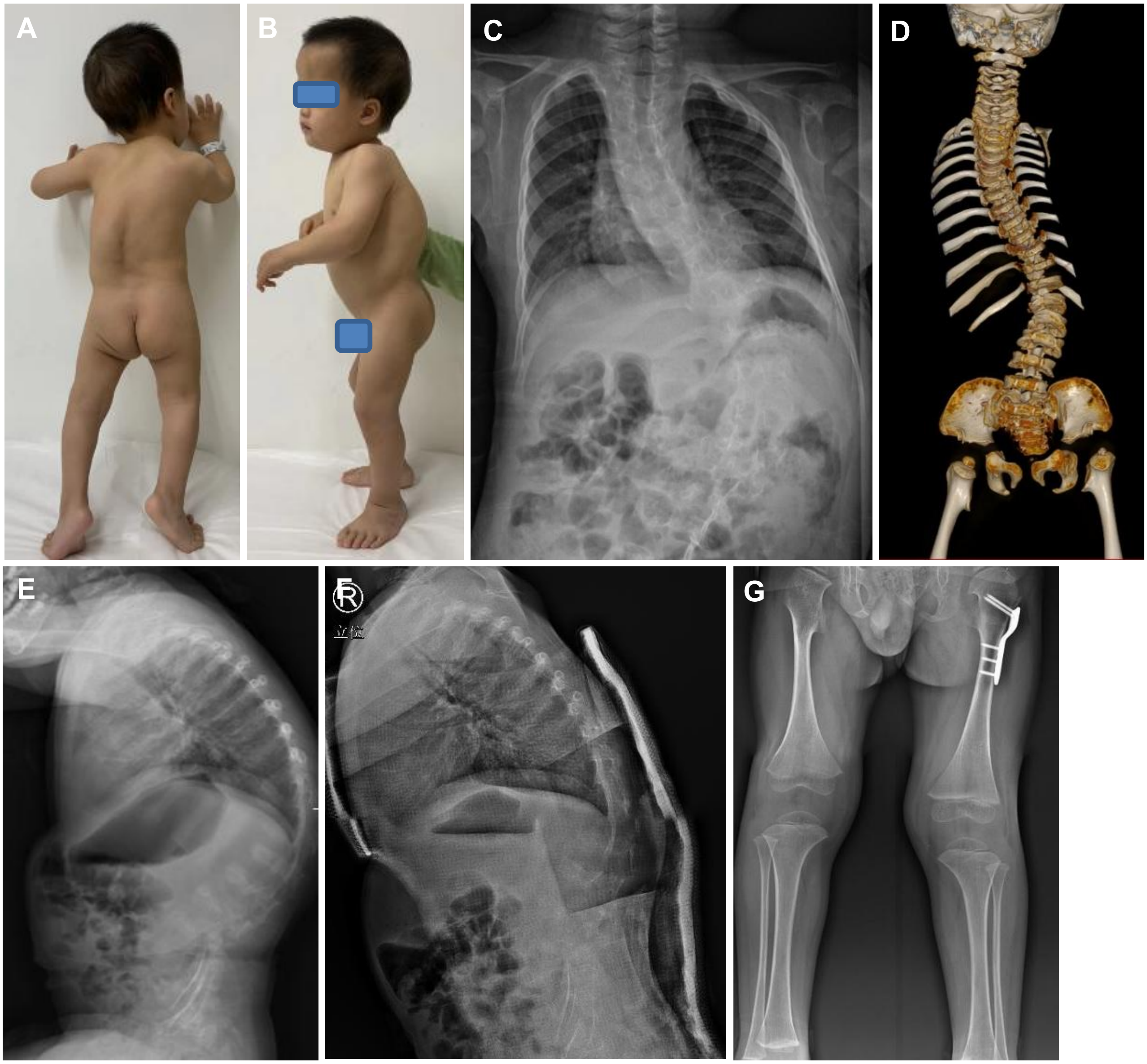

Clinical features of Case 7.

(A-B) Clinical photos of case 7. **(C-D)** Spine: X-ray and CT scan showed S-shaped scoliosis, thoracolumbar hyper-kyphosis, platyspondyly, overfaced pedicles. **(E-F)** Lateral view of the spine before and after body casting. **(G)** Long bones: dumbbell-shaped metaphyseal flaring.

Supplementary-Case 8

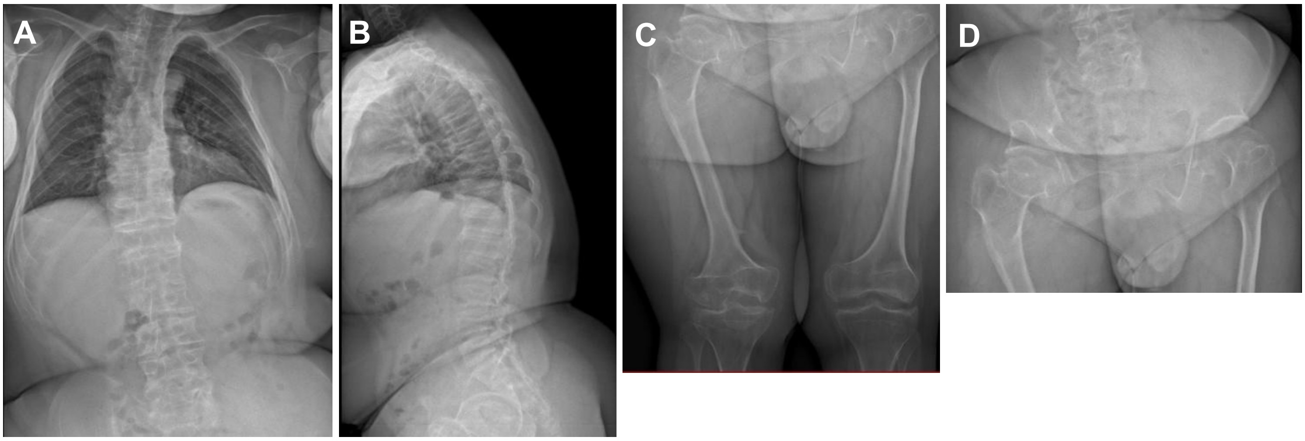

Clinical features of Case 8.

(A-B) Spine: mild scoliosis, mild kyphosis, platyspondyly and overfaced pedicles. **(C)** Long bones: dumbbell-shaped metaphyseal flaring, epiphyseal dysplasia, flattened femoral heads, shortened and thickened femoral necks. **(D)** Pelvis: mildly halberd-shaped pelvis.

Supplementary-Case 9

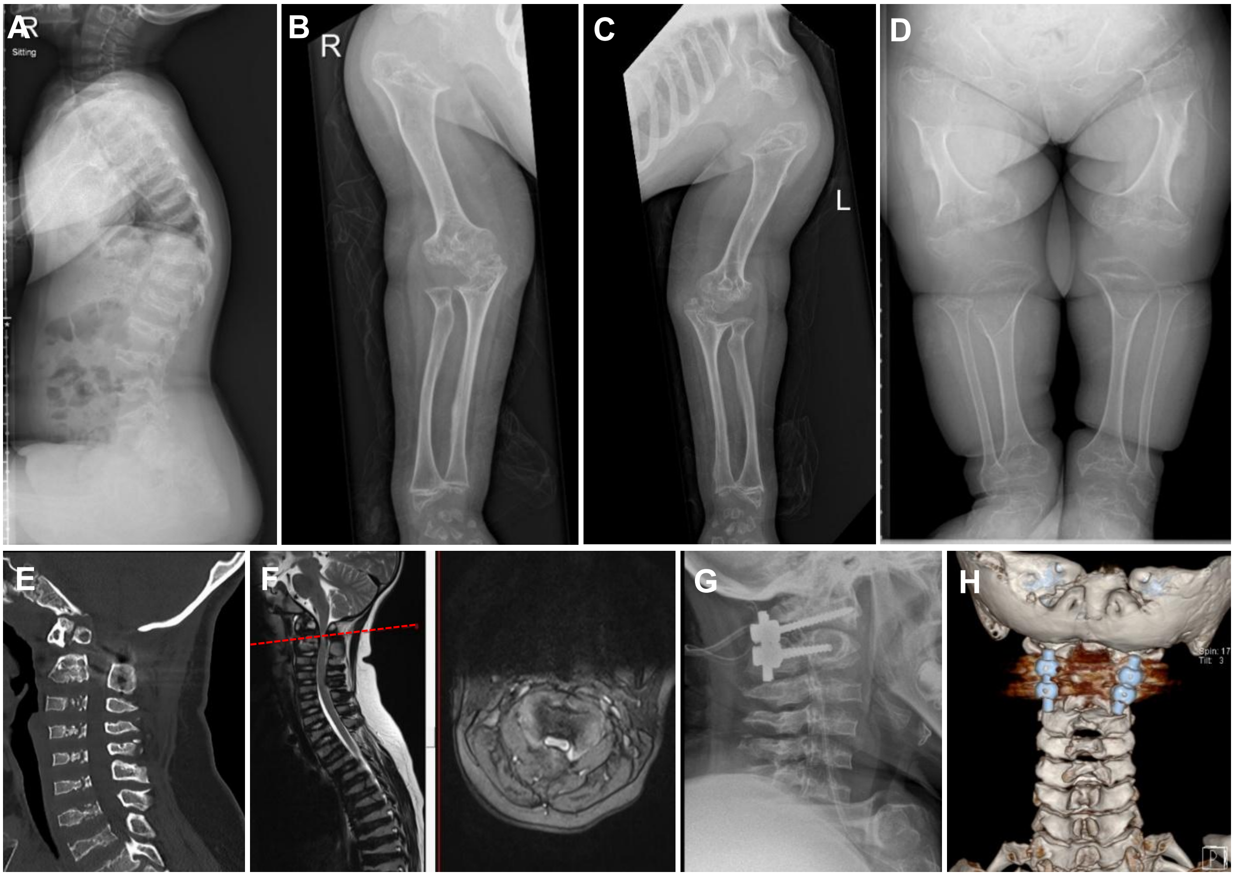

Clinical features of Case 9.

(A) Spine: thoracic hyper-kyphosis and lumbar lordosis. **(B-D)** Long bones: dumbbell-shaped metaphyseal flaring. **(E-F)** Atlantoaxial anterior translation with C1-C2 spinal canal stenosis. **(G-H)** Post-posterior atlantoaxial fusion.

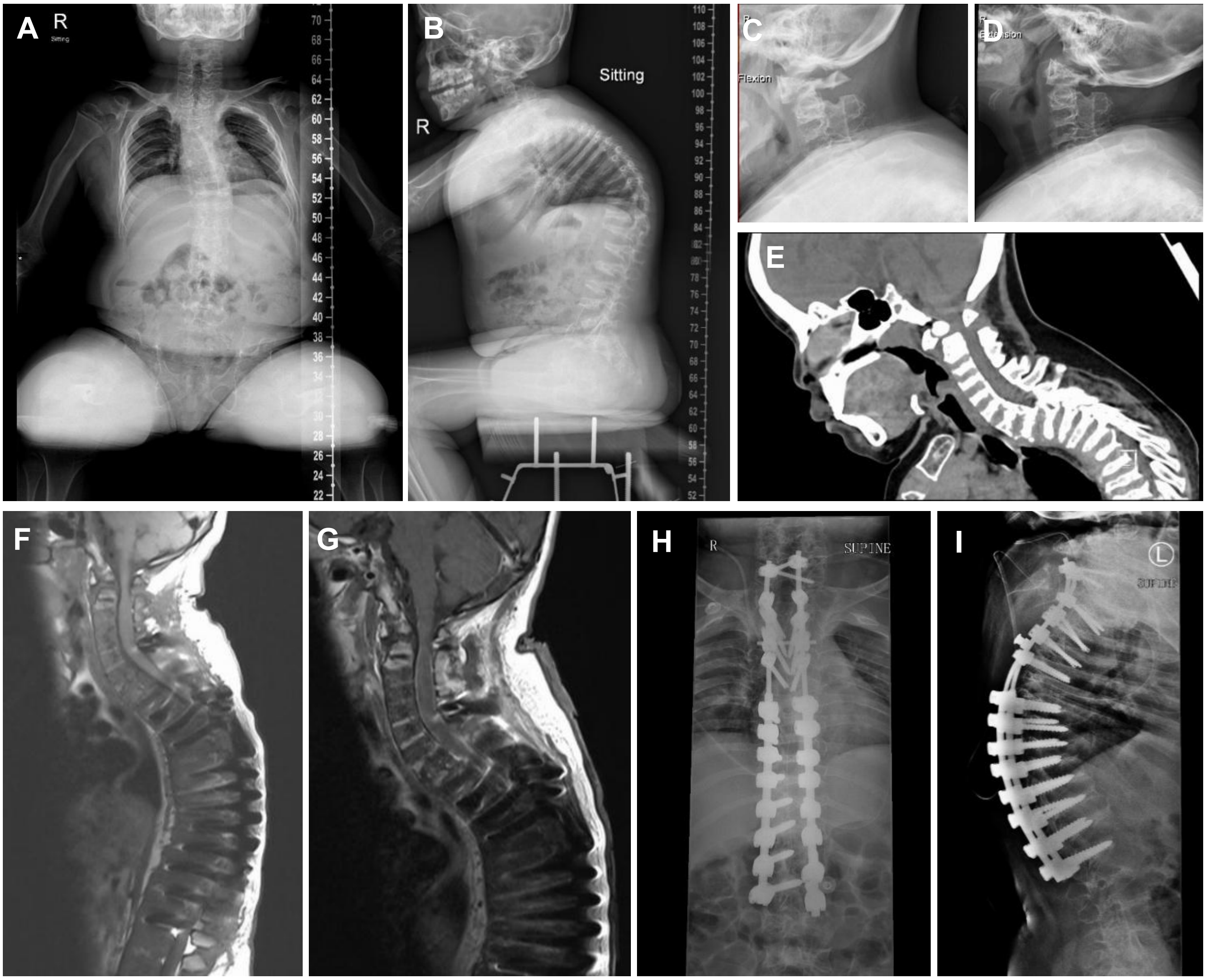

Clinical features of Case 10.

(A-B) Spine: S-shaped scoliosis, platyspondyly, overfaced pedicles. **(C-D)** Atlantoaxial instability with C1-C2 anterior displacement on flexion. **(E)** Posterior translation of the T2-T5 segments. **(F-I)** Post-spinal fusion.
